# Role of multi-modality therapy in peritoneal carcinomatosis and visceral metastasis: a case report and review of the literature

**DOI:** 10.1186/1477-7819-13-2

**Published:** 2015-06-30

**Authors:** Diego Vicente, Itzhak Avital, Alexander Stojadinovic

**Affiliations:** Department of Surgery, Walter Reed National Military Medical Center, 8901 Rockville Pike, Bethesda, MD 20889 USA; Bon Secours Cancer Institute, 6605 W Broad St, Richmond, VA 23230 USA

## Abstract

**Introduction:**

Treatment for advanced stage colorectal cancer with synchronous peritoneal carcinomatosis (PC) and hepatic metastasis (HM) has progressed significantly over the past 10 years.

**Case report:**

We present the case of a 39-year-old female patient with stage IV colorectal cancer with bilateral HM, pulmonary oligometastatic disease, and diffuse PC who underwent hyperthermic intraperitoneal chemotherapy (HIPEC) and complete cytoreductive surgery (CRS) for her intra-abdominal disease. The patient had an uneventful immediate post-operative recovery, and subsequently tolerated multiple cycles of adjuvant chemotherapy and percutaneous radiofrequency ablation of pulmonary lesions. At her 22-month follow-up assessment, the patient remains alive with disease.

**Conclusion:**

Current recommendations for surgical management of synchronous colorectal cancer PC and HM indicate that patients with less than three HMs, a low peritoneal cancer index (PCI), and good functional status will benefit most from CRS and HIPEC. Our patient had an elevated PCI of 12 as measured by computed tomography imaging, and five HMs (all less than 3 cm in size); however, given that her life expectancy on systemic chemotherapy was estimated to be approximately 12 months, we have observed carefully selected patients to benefit from an aggressive multi-modality approach. This case report demonstrates an all too common scenario for surgeons managing patients with advanced CRC, and highlights the importance of patient selection for surgical management as part of multidisciplinary cancer care in this patient population.

## Background

Over 100,000 patients were diagnosed with colorectal cancer (CRC) in 2012 in the United States, and it remains the second leading cause of cancer-related death [1]. Between 10 and 25% of patients with CRC are affected by peritoneal carcinomatosis (PC) [2, 3], and 35 to 55% will have hepatic metastasis (HM) during their disease course [4, 5]. While significant progress has been made in the independent management of HM and PC in CRC patients, the presence of HM in patients with known PC was traditionally considered a contraindication for cytoreductive surgery (CRS) [3, 6].

Recent studies have suggested a survival benefit for CRC patients with PC and HM with a combination of complete cytoreduction (CC0) or near complete cytoreduction (CC1) [7], hyperthermic intraperitoneal chemotherapy (HIPEC), and adjuvant systemic chemotherapy in carefully selected patients [8–15].

We present the case of a CRC patient with PC and HM who underwent multi-modality therapy consisting of CRS, HIPEC, adjuvant chemotherapy, and percutaneous radiofrequency ablation (RFA) of pulmonary lesions.

## Case presentation

**Figure 1:**
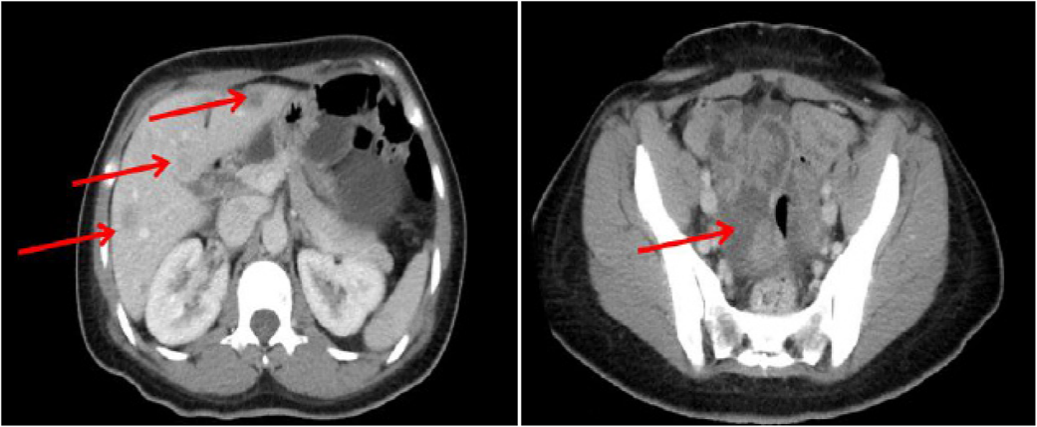
**Initial staging computed tomography scan at our institution demonstrating multiple hepatic metastasis and peritoneal carcinomatosis indicated by arrows.**

A 39-year-old, otherwise healthy, mother of four presented with intermittent lower abdominal pain. After diagnostic evaluation, she was found to have a partially obstructing sigmoid colon adenocarcinoma, likely metastatic hepatic lesions on her computed tomography (CT) scan, and elevated serum carcinoembryonic antigen (CEA; 461 ng/ml). The patient underwent a laparoscopic sigmoid colectomy and a biopsy of the omental peritoneal surface and left hepatic lesions in November 2012 at an outside institution. A pathologic evaluation of the resected colon specimen revealed moderately differentiated adenocarcinoma (pT4aN2aM1b) with lymphovascular and perineural invasion, disease in four of 12 involved mesenteric nodes, distant metastasis to the omentum, peritoneal surface, and liver, and positive mutation in codon 13 of the *K-ras* gene. Upon confirmation of peritoneal surface malignancy of colonic origin the patient was referred to our Surgical Oncology department for a consultation.Further evaluation at our center revealed a persistently elevated serum CEA (303 ng/ml), and cross-sectional imaging revealed multiple R > L hepatic lesions (five in total, all less than 3 cm in size), and peritoneal surface disease, with an estimated Peritoneal Cancer Index (PCI) score as measured by CT of 12 (Figure 1). Three separate 3-mm pulmonary nodules were also identified.

The patient was counseled extensively and given her primary concern to extend time with her family for any possible amount of time, her limited life expectancy if treated with systemic chemotherapy alone, and the resectable nature of her metastatic disease, the patient consented to proceed with multi-modality therapy consisting of CRS with HIPEC and pulmonary RFA, with intent to extend progression-free survival.

**Figure 2:**
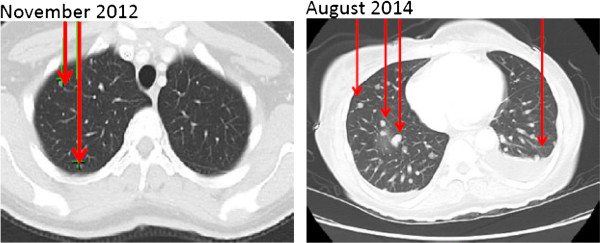
**Computed tomography scan images demonstrating progression of metastatic colorectal cancer pulmonary disease indicated by arrows from the patient’s initial staging work up at our institution through her transition to aggressive comfort care.**

In December 2012 the patient underwent CRS with multi-visceral resection (right hepatectomy, wedge resection of the left liver, omentectomy, splenectomy, subtotal colectomy, hysterectomy, bilateral salpingo-oophorectomy, retroperitoneal lymphadenectomy, partial cystectomy, and peritonectomy), and clearance of all grossly apparent intra-peritoneal disease (CC0). After cytoreduction we used bi-directional intraoperative chemotherapy consisting of intravenous 5-fluorouracil and leucovorin, in conjunction with oxaliplatin-based HIPEC, over 35 minutes at a temperature of 42°C.On post-operative day seven, the patient was discharged home. Her immediate post-operative course was uneventful. The patient tolerated six cycles of leucovorin, fluorouracil, and oxaliplatin (FOLFOX) adjuvant systemic therapy with good response, as well as percutaneous CT-guided RFA of the metastatic pulmonary lesions in post-operative month five. In post-operative month nine, the patient presented with a small bowel obstruction secondary to a diaphragmatic hernia. The diaphragmatic hernia was repaired, and a single retroperitoneal metastatic recurrence was identified and ablated during this procedure. The patient maintained excellent performance status (Eastern Cooperative Oncology Group (ECOG) 0) throughout this portion of her care, though she demonstrated progression of her pulmonary disease in post-operative month 10 and received an additional seven cycles of palliative systemic FOLFOX therapy. Progression of the disease was again noted in post-operative month 13 and the patient was treated with pelvic radiation for a sacral metastasis, RFA for pulmonary lesions, and leucovorin, fluorouracil, and irinotecan (FOLFIRI) systemic therapy. The patient was transitioned to 5-fluorouracil and bevacizumab, due to irinotecan-related toxicity, in post-operative month 17. Though there was no further evidence of intra-abdominal metastatic disease, the patient’s pulmonary disease progressed on this palliative systemic therapy over a four-month period (Figure 2) and the patient then transitioned to aggressive comfort care in post-operative month 22.

## Conclusion

We present a patient with CRC with peritoneal surface and visceral spread of the disease to the liver and lung. Our management options for this patient included: (1) palliative care; (2) systemic chemotherapy alone; or (3) aggressive multi-modality therapy with curative intent, consisting of CRS with HIPEC and systemic chemotherapy, as well as pulmonary lesion ablation and/or resection.

Patients with peritoneal surface malignancy of colorectal origin and HM are carefully selected for CRS and HIPEC based on a thorough review of comorbidities, functional status (ECOG), extent of peritoneal surface involvement (PCI), number, size, and distribution of HM, as well as the ability to achieve complete resection and/or ablation of disease [8, 14]. Appropriate timing of CRS and systemic therapy is also important for these patients. At this time, neoadjuvant chemotherapy for resectable HM [16, 17] or PC [18, 19] from CRC has demonstrated mixed results in the literature. Determination of optimal timing of treatment should be made based on a multidisciplinary approach, particularly in cases of resectable synchronous HM, PC, and pulmonary metastasis of CRC origin.

Our patient was young, in excellent health, and with excellent performance status at the time of presentation. Her PCI and size and number of hepatic and pulmonary metastasis made her a suitable candidate for consideration of multi-modality therapy with curative intent. Estimated survival with modern systemic chemotherapy alone is less than 13 months [3, 20–23], whereas a median survival of approximately 20 months (with a five-year overall survival rate of approximately 30% may be achieved in selected patients that undergo optimal cytoreduction in conjunction with regional chemotherapy.

Several reports have demonstrated the survival benefit of CRS and HIPEC for CRC patients with combined PC and HM [8–14], and a meta-analysis of these trials revealed that CRS and HIPEC offers improved survival compared to the expected survival with modern systemic chemotherapy [15]. Three of these studies reported on prognostic factors for survival after CRS and intraperitoneal chemotherapy, as seen in Table 1. Similar to CRC patients with only PC who undergo CRS, poor prognostic factors for patients with PC and HM who undergo surgery with curative intent include high PCI (or Gilly classification) and inadequacy of resection. The largest series (n = 37) from these studies noted that patients with a PCI ≥12 or three or more HMs had a median overall survival of 27 months, which was significantly shorter than those with a PCI <12 and one to two HMs (median survival of 40 months) [14]. Of the three patients in this study who, like our patient, had a PCI ≥12 and three or more HMs, and required a major liver resection, one patient died within the first postoperative month, while the remaining two patients survived to between 25 and 36 months. Other studies have also demonstrated that patients with an elevated PCI who undergo liver resection and adequate resection of intra-abdominal disease (CC0 or CC1) who do not succumb to operative mortality (during the first postoperative month) can achieve survival for more than 13 months [8, 11].

**Table 1:** **Review of literature demonstrates four studies which evaluated prognostic factors for colorectal cancer patients with peritoneal carcinomatosis (PC) and hepatic metastasis (HM)**

Article	Number of patients with PC and HM in study	PCI mean +/-SD (range)	Gilly classification (% of subjects)	Number of median HM	Number of simultaneous major liver resections	Number (%) of patients with CC0/CC1 or R0/R1	Overall survival (months)	Poor prognostic factor 1	Poor prognostic factor 2	Poor prognostic factor 3	Poor prognostic factor 4	Findings
Carmignani 2004 [8]*	16	-	-	-	-	15 (55%)	15	PCI ≥13	>2.5 mm of residual disease	-	-	No difference in survival comparing HM to PC patients
Kianmanesh 2007 [10]	16	-	III (14%) IV (63%)	-	3	30 (70%)	36	Gilly 3 or 4	>5 mm of residual disease	-	-	Addition of HM resection to PC treatment did not influence survival compared to PC treatment alone.
Varbaan 2009 [12]	14	-	-	1	2	9 (64%)	23	No prognostic factors identified on univariate analysis.	-	-	-	
Maggiori 2013 [14]	37	11 (1-26)	-	6	12	37 (100%)	32	PCI ≥12	LN status of primary cancer	No postoperative systemic chemotherapy	Synchronous resection of PC and HM	Prolonged survival can be achieved with CRS and HIPEC in patients with PCI <12 and HM <3.

As seen in studies evaluating CRS and HIPEC in PC patients only, the principal prognostic factors (1, 2, 3, 4) for patients with PC and HM were extent of peritoneal involvement and adequacy of resection.

^*^Though the majority of patients had both PC and HM, the authors did not differentiate patients with PC and HM from patients with PC and extra-hepatic metastasis in this study.

- Value not reported.

CC0: Complete Cytoreduction; CC1: near complete cytoreduction; CRS: Cytoreductive Surgery, HIPEC: Hyperthermic Intraperitoneal Chemotherapy; HM: Hepatic Metastasis, PC: Peritoneal Carcinomatosis, PCI: Peritoneal Carcinomatosis Index, LN: Lymph Node.

Beyond survival, a second major concern for patients with PC and HM is the potential morbidity from the addition of a major procedure to CRS. Interestingly, two reports have demonstrated that the addition of hepatic resection in this setting has not significantly increased the morbidity compared to CRS for PC alone [12, 14]. In the case of our patient, she developed a left-sided diaphragmatic hernia after CRS, which was repaired in postoperative month nine, and she subsequently had no further intra-abdominal complications from the CC0 of her extensive intra-abdominal pathology.

At this time, the patient has been transitioned to comfort care; however, she is thankful for the time she was able to enjoy with her family (her primary concern) and is alive with the disease at 22 months from her diagnosis, which is beyond her estimated survival on systemic chemotherapy alone. While emerging studies are beginning to define which metastatic CRC patients will benefit most from CRS, the overall number of patients in these studies remains limited (n <100). Hence patient selection in conjunction with multidisciplinary consultation, thorough informed consent, and careful consideration of ‘name the disease, stage the disease, assess resectability, and determine operability’ by a capable team with extensive experience with CRS and HIPEC are imperative.

### Consent

Consent was obtained from the patient’s family for publication of this case report.

## Abbreviations

PC: Peritoneal Carcinomatosis
HM: Hepatic Metastasis
HIPEC: Hyperthermic Intraperitoneal Chemotherapy
CRS: Cytoreductive Surgery
PCI: Peritoneal Cancer Index
CRC: Colorectal cancer
CC0: Complete Cytoreduction
CC1: Near complete cytoreduction
RFA: Radiofrequency Ablation
CT: Computed Tomography
CEA: Carcinoembryonic antigen
FOLFOX: Leucovorin, Fluorouracil, and Oxaliplatin
ECOG: Eastern Cooperative Oncology Group
FOLFIRI: Leucovorin, Fluorouracil, and Irinotecan.
